# Systematic Review of Prevalence of *Histoplasma* Antigenuria in Persons with HIV in Latin America and Africa

**DOI:** 10.3201/eid3008.231710

**Published:** 2024-08

**Authors:** Preethiya Sekar, Gila Hale, Jane Gakuru, David B. Meya, David R. Boulware, Jayne Ellis, Elizabeth Nalintya, Nathan C. Bahr, Radha Rajasingham

**Affiliations:** University of Minnesota, Minneapolis, Minnesota, USA (P. Sekar, D.R. Boulware, N.C. Bahr, R. Rajasingham);; Makerere University Infectious Diseases Institute, Kampala, Uganda (G. Hale, J. Gakuru, D.B. Meya, E. Nalintya);; London School of Hygiene and Tropical Medicine, London, UK (J. Ellis);; University of Kansas, Kansas City, Kansas, USA (N.C. Bahr)

**Keywords:** histoplasmosis, HIV/AIDS and other retroviruses, advanced HIV disease, sexually transmitted infections, fungi, fungal infections, retroviruses, viruses, antigenuria, prevalence, Latin America, the Americas, Africa

## Abstract

Histoplasmosis is a fungal disease associated with substantial mortality rates among persons with advanced HIV disease. Our systematic review synthesized data on the global prevalence of *Histoplasma­*-caused antigenuria in persons with HIV. We searched PubMed/Medline, Embase, and Scopus databases on January 3, 2023, to identify cross-sectional and cohort studies evaluating *Histoplasma* antigenuria prevalence among adults with HIV infection. We calculated point estimates and 95% CIs to summarize prevalence. Of 1,294 studies screened, we included 15. We found *Histoplasma* antigenuria among 581/5,096 (11%; 95% CI 11%–12%) persons with HIV and 483/3,789 persons with advanced HIV disease (13%; 95% CI 12%–14%). Among persons with HIV and symptoms consistent with histoplasmosis, *Histoplasma* antigenuria prevalence was 14% (95% CI 13%–15%; 502/3,631 participants). We determined that persons with advanced HIV disease, inpatients, and symptomatic persons might benefit from a systematic approach to early detection of histoplasmosis using urine antigen testing.

Histoplasmosis is an endemic fungal infection caused by *Histoplasma capsulatum*. In immunocompetent persons, histoplasmosis is often asymptomatic or localized to the pulmonary system. However, in immunocompromised persons, histoplasmosis can manifest in a progressive disseminated form that is an AIDS-defining illness. Even with prompt treatment, disseminated histoplasmosis has a high mortality rate (*1*,*2*). Given historically limited awareness of the disease and poor diagnostic capacity, histoplasmosis has been underdiagnosed, and the true global burden remains unknown. Although well documented in much of the Americas, with recent studies showing prevalence rates up to 30% in some areas of Central America (*3*), histoplasmosis is also considered endemic in parts of Africa and Asia, and expanding regions of endemicity have been recognized in the past decade (*4*). Given the potential illness and death from HIV-associated histoplasmosis, a critical need exists for improved diagnostics and treatments. 

In persons with HIV, histoplasmosis can be difficult to detect because the signs and symptoms of disseminated histoplasmosis closely mimic those of disseminated tuberculosis (TB), increasing risk of misdiagnosis and undertreatment, especially in regions where both diseases are endemic (*5*). Histopathology and culture provide the most accurate methods for diagnosing histoplasmosis, but resource-limited settings often lack the personnel and laboratory infrastructure to use these methods.

Enzyme immunoassays (EIAs), where available, have bridged this diagnostic gap by enabling detection of *Histoplasma* antigen in both urine and serum samples (*6*). The first EIA test, developed by MiraVista Diagnostics (https://miravistalabs.com), is 95% sensitive in urine samples (*7*), but this test is not a viable option for low- and middle-income countries because samples can be processed only at 1 reference laboratory in Indianapolis, Indiana, USA. Optimum Imaging Diagnostics (https://optimumimaging.com) has also developed a urine histoplasmosis sandwich EIA using rabbit monoclonal antibodies that has a sensitivity of 92% and a specificity of 32% (*8*). The IMMY Alpha test (https://www.immy.com) is a US Food and Drug Administation–approved polyclonal antibody ELISA that has 62%–81% sensitivity and 96%–97% specificity (*7*,*9*). IMMY subsequently developed the Clarus *Histoplasma* galactomannan assay, a monoclonal antibody test, for use on urine samples; sensitivity is 91% and specificity is 91% (*7*). Advent of urinary EIA screening tests might offer a feasible testing strategy, especially in endemic settings, for earlier detection of active histoplasmosis infection than with other testing strategies (*10*). The historical absence of sufficiently sensitive rapid testing that can be routinely performed in resource-limited settings has led to a dearth of accurate prevalence data on the global burden of histoplasmosis. 

By performing this systematic review, we aimed to synthesize current research about the prevalence of *Histoplasma* antigens in urine (antigenuria) among persons living with advanced HIV disease worldwide. Understanding the prevalence and burden of *Histoplasma* antigenuria might aid in developing screening and treatment algorithms to improve clinical outcomes related to HIV-associated histoplasmosis. We registered our study at PROSPERO International prospective register of systematic reviews (study identification CRD42023399523).

## Methods

### Eligibility Criteria

We included cross-sectional or cohort studies of adults living with HIV in whom *Histoplasma* antigen testing was performed. We included studies conducted in inpatient and outpatient settings that had both asymptomatic and symptomatic participants. We defined symptomatic histoplasmosis as having >1 associated clinical signs and symptoms, such as fever, weight loss, night sweats, and respiratory symptoms. We excluded studies involving nonhuman subjects or persons <18 years old, non-*Histoplasma* studies, non–English-language studies, conference abstracts, reviews, case reports, and commentaries. 

### Search Strategy

**Figure 1:**
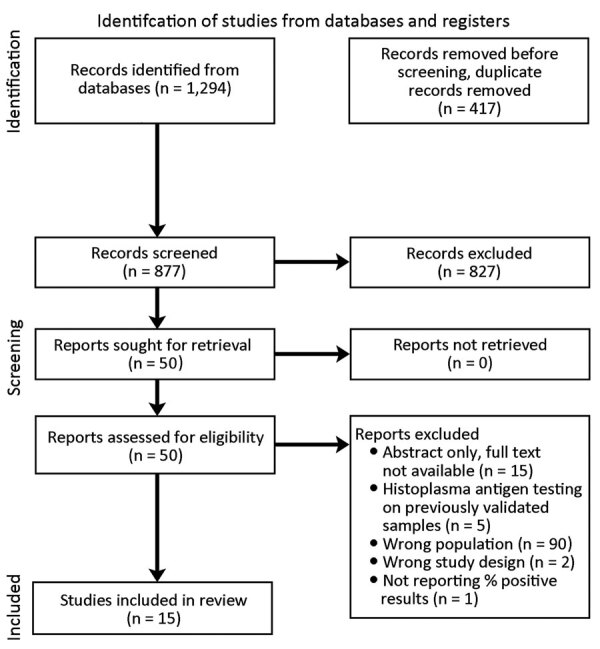
PRISMA (Preferred Reporting Items for Systematic Reviews and Meta-Analyses) flow diagram for systematic review of prevalence of *Histoplasma* antigenuria in persons with HIV in Latin America and Africa.

We conducted a systematic search of PubMed/Medline, Embase, and Scopus databases using PRISMA (Preferred Reporting Items for Systematic Reviews and Meta-Analyses; https://www.prisma-statement.org) guidelines for studies published during January 1, 1947–January 3, 2023 (Figure 1). Search terms included combinations of histoplasmosis, antigen detection, and HIV or advanced HIV. We updated the search on July 20, 2023 (Appendix Table 1). 

### Data Collection

Three systematic review team members independently assessed abstracts to select articles for full-text review based on specified criteria using Rayyan (https://www.rayyan.ai) and resolved disagreements through discussion. For the full-text review, we used a Qualtrics form (https://www.qualtrics.com) to abstract data. Three reviewers independently completed the form, and a fourth adjudicated discrepancies. Data abstracted were journal name and publication date, article title, first author name, study design, geographic location, setting (inpatient or outpatient), clinical phenotype (i.e., screening among asymptomatic or symptomatic persons according to study author definitions), age range, sex, percentage of participants on antiretroviral therapy, median CD4 count, active TB infection (presumed versus confirmed), type of *Histoplasma* antigen testing kit, other *Histoplasma* diagnostics employed, sample size, number of positive *Histoplasma* cases, percentage of participants with *Histoplasma* detected, and 95% CIs. We also reviewed treatment provided, prophylaxis, and clinical outcomes, but that information was not consistently available across studies. 

### Outcome Measures

We assessed *Histoplasma* antigen prevalence among subgroups, including persons with HIV regardless of CD4 count, persons with advanced HIV disease (CD4 <200 or World Health Organization stage 3 or 4), inpatients, outpatients, patients with symptoms of histoplasmosis (i.e., fevers, weight loss, night sweats, respiratory symptoms), and asymptomatic patients. 

### Risk of Bias Assessment

We determined the risk for bias for cross-sectional studies using the Agency for Healthcare Research and Quality Methodology Checklist (Appendix Table 2) and for cohort studies using the modified criteria of the Risk of Bias Assessment Tool for Nonrandomized Studies (Appendix Table 3). The cross-sectional studies checklist assesses the presence, absence, or undetermined status of 11 items: sources of data, inclusion/exclusion criteria, time period, study population selection, blinding, quality assurance of testing, reason for patient exclusion, missing data, confounding, completeness of data collection, and follow-up for missing data (Appendix Table 5). The cohort study assessment tool rates risk for bias as low, high, or unclear in 6 different domains: participant selections, confounding variables, measurement of exposures, blinding of outcome assessments, incomplete outcome data, and selective outcome reporting. Three reviewers independently determined the risk for bias in each domain for each eligible study; a fourth reviewer identified and resolved any disagreements. We classified included studies at low risk of bias if >4 of 6 domains were determined to be low. We classified remaining studies at high risk of bias. 

### Effect Measures and Synthesis Methods

We calculated point estimates and 95% CIs for each of the study outcomes. We also stratified studies selected for full review and data extraction for subgroup analyses on the basis of attributes (e.g., inpatient cohort vs. symptomatic cohort). If inpatient versus outpatient setting was not specified in an article, or if disaggregated data were not provided, we excluded those studies from the setting subgroup analysis. We included studies in the symptomatic cohort if they described participants with >1 clinical signs: fever, weight loss, night sweats, or respiratory symptoms. We grouped participants without any signs or symptoms at time of screening in the asymptomatic cohort. If cohorts included a mixture of symptomatic and asymptomatic participants, only those studies that provided disaggregated prevalence results stratified by symptom status were included in the subgroup analysis. We pooled estimates with 95% CIs based on subgroups for forest plots and summary tables. We used Microsoft Excel 2019 version 16.7 (https://www.microsoft.com) when conducting analyses. We used the GRADE (Grading of Recommendations, Assessment, Development, and Evaluations) approach to assess the certainty of evidence by considering risk of bias, inconsistency, indirectness, imprecision, and publication bias (*11*). Four levels of certainty ratings were possible: very low, low, moderate, and high. 

## Results

### Study Selection

Through our database search we identified 1,294 titles. After deduplication, 877 titles remained for abstract review. Of the 877 abstracts reviewed, we excluded 827 because they were not published in English (n = 9), were case reports (n = 416) or systematic reviews (n = 38), did not have a study population with confirmed HIV infection status (n = 36), did not use *Histoplasma* antigen testing (n = 129), or did not report the percentage of the cohort that was *Histoplasma* antigen positive (n = 199). Of the remaining 50 publications available for full-text review, we excluded 35 (Figure 1). 

### Study Characteristics

**Figure 2:**
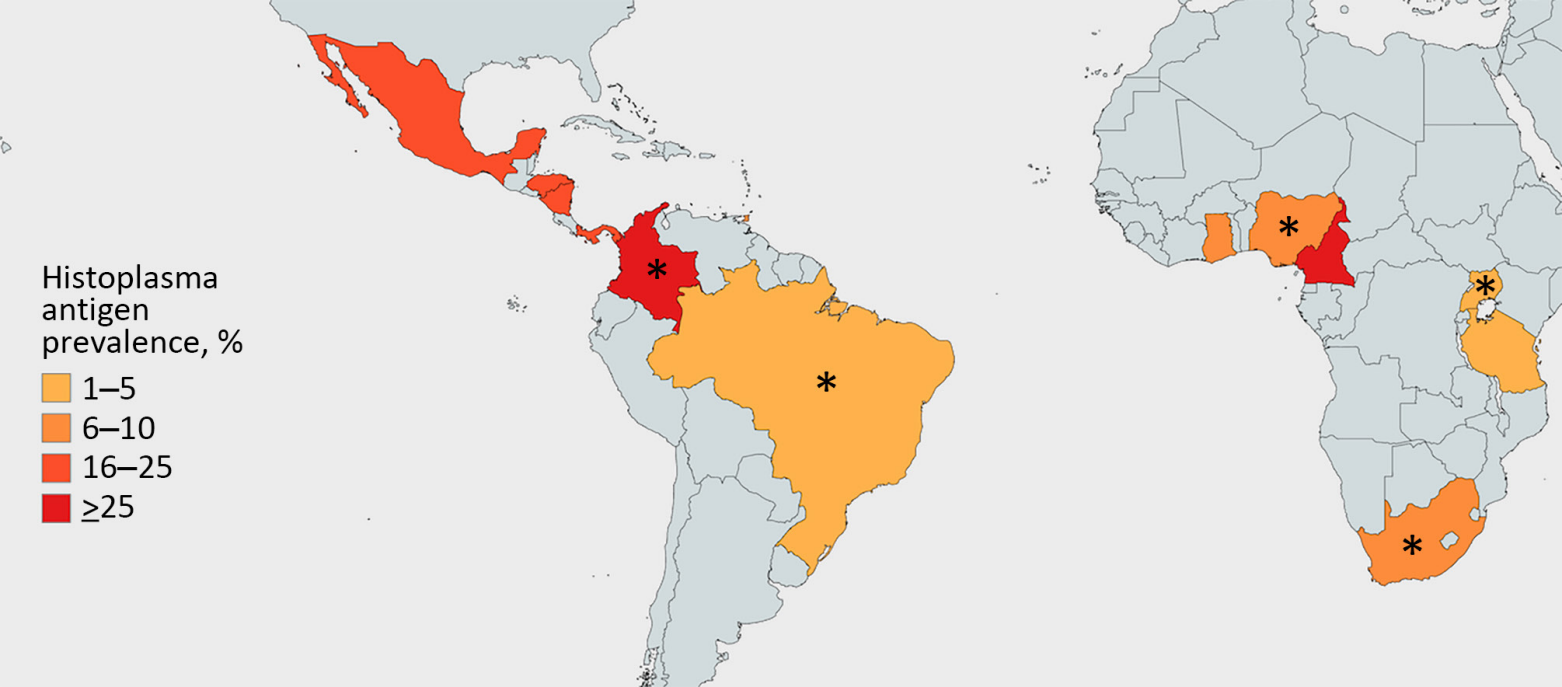
Country-level *Histoplasma* antigenuria prevalence in systematic review of prevalence of *Histoplasma* antigenuria in persons with HIV in Latin America and Africa. Asterisks denote countries with studies that were done in advanced HIV populations, whereas solid colors denote countries with studies of participants with HIV screened for histoplasmosis irrespective of CD4 count.

We included 15 articles published during 2012–2023 covering 5,096 HIV-positive adults (cohort sizes 35–4,453) undergoing urinary *Histoplasma* antigen testing. Eleven studies (n = 4,057/5,096 participants) were cross-sectional and the other 4 were prospective cohort studies (Table). Nine cross-sectional studies (2,753/5,096 participants) took place in sub-Saharan Africa; the remaining 2 were performed in Central or South America (2,343/5,096 participants) (Figure 2). No studies evaluated *Histoplasma* antigenuria prevalence in Asia, Europe, Australia, or North America. Ten of 15 studies (3,789/5,096 participants) focused specifically on populations with advanced HIV disease, whereas 5 studies included all persons with HIV, regardless of CD4 count or World Health Organization disease stage. Eight studies (3,631/5,096 participants) described persons who initially sought treatment with symptoms, and 7 studies (1465/5096 participants) focused on asymptomatic screening cohorts. Most studies were conducted in the outpatient setting, but 8 recruited hospitalized patients (3,286/5,096 participants). The 15 included studies used a mixture of tools to identify *Histoplasma* antigenuria: 2 used MiraVista *Histoplasma* Quantitative EIA test, 4 used IMMY Alpha *Histoplasma* EIA, 7 used IMMY GM *Histoplasma* EIA, 1 used Optimum Imaging Diagnostics *Histoplasma* Sandwich EIA, and 1 used ELISA. 

**Table:** Characteristics of *Histoplasma* urine antigen prevalence studies included in systematic review of prevalence of *Histoplasma* antigenuria in persons with HIV in Latin America and Africa*

Reference	Country	Study design	Study population†	Clinical status	Setting	Test type‡	No. participants	No. samples tested	HUA positivity, %
(*14*)	Tanzania	Cross-sectional	All HIV and non-HIV	Symptomatic	Inpatient	MV EIA	628	628	1.1
(*15*)	Uganda	Cohort	Advanced HIV	Asymptomatic	Outpatient	IMMY GM EIA	388	388	1.0
(*16*)	Uganda	Cohort	Advanced HIV/meningitis	Symptomatic	Inpatient	MV EIA	257	257	0.0
(*17*)	South Africa	Cross-sectional	Advanced HIV	Symptomatic	Outpatient	IMMY Alpha EIA	34	17	23.5
(*18*)	South Africa	Cross-sectional	Advanced HIV	Asymptomatic	Inpatient	IMMY GM EIA	189	189	5.8
(*19*)	Cameroon	Cross-sectional	All HIV	Asymptomatic	Outpatient	OIDx EIA	138	138	26.1
(*20*)	Ghana	Cross-sectional	All HIV	Asymptomatic	Outpatient	IMMY GM EIA + OIDx LFA	150	107	5.6
(*21*)	Nigeria	Cross-sectional	Advanced HIV	Symptomatic	Outpatient	IMMY GM EIA	213	41	7.3
(*22*)	Nigeria	Cross-sectional	Advanced HIV	Symptomatic	Inpatient	IMMY GM EIA	988	988	7.7
(*23*)	Mexico	Cohort	Advanced HIV	Symptomatic	Inpatient	IMMY Alpha EIA	288	288	21.5
(*24*)	Brazil	Cohort	Advanced HIV	Asymptomatic	Inpatient	IMMY Alpha EIA	106	106	3.8
(*25*)	Colombia	Cross- sectional	Advanced HIV	Symptomatic	Inpatient	IMMY Alpha EIA	172	172	29.1
(*26*)	Colombia	Cross-sectional	Advanced HIV	Asymptomatic	Outpatient	CDC ELISA	768	154	20.1
(*27*)	Panama, Honduras, Nicaragua	Cross-sectional	All HIV	Symptomatic	Inpatient	IMMY GM EIA	4453	1343	20.0
(*28*)	Trinidad	Cross-sectional	HIV	Asymptomatic	Outpatient	IMMY GM EIA + OIDx LFA	280	280	6.4

*EIA, enzyme immunoessay; GM, galactomannan; HUA, histoplasma urine antigen; LFA, lateral flow assay. †All HIV refers to participants with HIV who were not stratified by CD4 or limited to CD4 counts <200, such as persons in advanced HIV cohorts. ‡Test manufacturers and types: MV, MiraVista Diagnostics EIA (https://miravistalabs.com); IMMY Alpha and GM EIAs (https://www.immy.com); OIDx, Optimum Imaging Diagnostics LFA (https://optimumimaging.com).

### *Histoplasma* Antigen Prevalence

**Figure 3:**
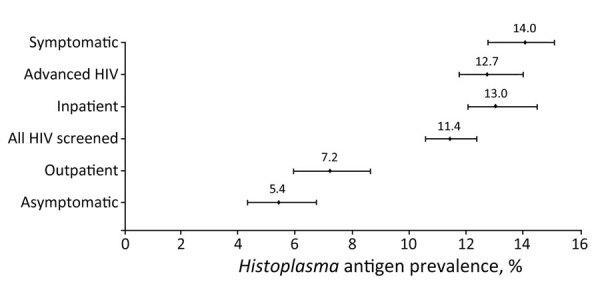
Forest plot of *Histoplasma* antigen prevalence among subgroups of interest in systematic review of prevalence of *Histoplasma* antigenuria in persons with HIV in Latin America and Africa. Error bars indicate 95% CIs.

Of 5,096 persons with HIV tested for *Histoplasma* antigen in their urine from the 15 included studies, 11% (95% CI 11%–12%; n = 581) were antigen positive. Among persons with advanced HIV disease, prevalence of *Histoplasma* antigenuria was 13% (95% CI 12%–14%; n = 483) (Figure 3). Among symptomatic persons with HIV, 14% (95% CI 13%–15%; n = 502) were antigen positive, whereas among asymptomatic persons, 5% (95% CI 4%–7%; n = 79) were *Histoplasma* antigen positive. *Histoplasma* antigen prevalence among inpatients with HIV was 13% (95% CI 12%–14%; 433/3,286) and among outpatients was 7% (95% CI 6%–9%; 111/1,549) (p<0.05). We also noted marked geographic variation, with the prevalence of both *Histoplasma* antigenuria and histoplasmosis ranging from 1% in Uganda to 26% in Cameroon (Table; Figure 2). 

### Risk of Bias in Studies

All of the included studies were observational in nature (Appendix Table 4). In the 2 cohort studies, we found uncertain risk of bias in the selective outcome reporting and low risk throughout all other domains. We determined that all 13 cross-sectional studies defined their information sources, listed inclusion and exclusion criteria for participant selection, indicated at least the start date of the testing period, and specified populations. Two of 13 studies were blinded; the remaining 11 studies did not specify blinding. Six of 13 studies had persons excluded from analyses, often because the participant lacked urine samples. Seven studies addressed confounding variables such as TB or other opportunistic infections; the remaining 8 studies did not test participants for confounding conditions. Furthermore, thorough analysis of bias risk is consistent with overall low risk of bias, although some categories of risk were unclear in individual studies. 

## Discussion

From 15 studies that assessed the prevalence of *Histoplasma* antigenuria among 5,096 persons with HIV, we identified 11% (95% CI 11%–12%) *Histoplasma* antigenuria prevalence among all persons with HIV and 13% (95% CI 12%–14%) among persons with advanced HIV disease. Among symptomatic persons with HIV, *Histoplasma* antigenuria prevalence was 14% (95% CI 13%–15%), whereas among asymptomatic persons with HIV, prevalence was 5% (95% CI 4%–7%). Taken together, those data help highlight populations that might benefit from systematic screening for histoplasmosis as part of an HIV package of care. Our analysis suggests that the highest yield screening programs would likely be among persons with advanced HIV disease, histoplasmosis inpatients, and persons who have symptoms consistent with histoplasmosis. Whereas we noted general trends in *Histoplasma* antigenuria prevalence by population characteristics, in some individual studies, those trends did not hold true (e.g., low histoplasmosis prevalence among inpatients with advanced HIV). Various factors aside from inpatient/outpatient status and presence of signs and symptoms might explain this variation, such as geographic location, underlying patient characteristics, and the nonspecific nature of symptoms associated with disseminated histoplasmosis that are shared with other conditions. Therefore, summary data must be considered in the context of the population characteristics of individual studies. 

Our systematic review was limited by the modest number of studies available, but the moderate size of our cohorts provided good precision around our prevalence estimates. Included prevalence studies came from just 6 countries in Central and South America and 9 in sub-Saharan Africa, but few studies from Asia, North America, Europe, and Australia have been published. In our analysis, we found only 3 countries (South Africa, Nigeria, and Colombia) in which >1 antigen prevalence study had been conducted. Prevalence of histoplasmosis transmission is classically thought to be associated with sources for local exposure, such as bat guano, poultry, caves, housing, construction, and certain occupations, although persons not reporting those classic exposures can also manifest histoplasmosis. This duality underscores the importance of conducting regional prevalence studies to assess fluctuations in histoplasmosis prevalence. Prospective studies are needed to better understand disease prevalence and consider the effects of symptoms, CD4 count, coexisting TB infection, and TB diagnostics on recognizing the disease. 

The observational nature of all of the included studies and resulting selection bias limits the generalizability of our findings; inpatient studies might have excluded TB patients before enrollment. For example, in studies of identified *Histoplasma* antigenuria among only symptomatic inpatients, prevalence might have been higher than among inpatients in whom TB had not yet been excluded. In cohort studies, there might be a bias to include persons with confirmed histoplasmosis and consequentially report a higher prevalence of histoplasmosis compared with cross-sectional screening studies. Very few studies included TB diagnostics or consideration of coexisting TB infection. The small number of studies and limited data also prevented any further subgroup analyses of *Histoplasma* antigenuria prevalence (e.g., by antiretroviral therapy status). Prospective clinical studies are needed to identify persons with advanced HIV disease who have asymptomatic *Histoplasma* antigenuria, particularly those from geographic areas not represented by currently available data. 

*Histoplasma* antigenuria prevalence among asymptomatic persons with HIV was 5%. The clinical significance of asymptomatic *Histoplasma* antigenuria among persons with HIV is unknown, and further research is required to investigate whether antifungal therapy is required or a watch-and-wait policy alongside antiretroviral therapy with immune reconstitution is sufficient among persons in that group. Those results could be false positives; they could indicate that patients with a low antigen burden who are undergoing antiretroviral therapy might not require antifungal therapy; or they could represent early dissemination that would progress without antifungal treatment. 

Clinical studies and studies on the burden of histoplasmosis disease are hampered by a historical lack of histoplasmosis diagnostics. Although culture and histopathology are the most accurate methods for diagnosis, many resource-limited settings do not have the laboratory capacity or staffing to perform these tests. Furthermore, culture results require several weeks, which is clinically impractical. Antigen detection assays for histoplasmosis have 95% sensitivity (95% CI 94%–97%) and 97% specificity (95% CI 97%–98%) and are found to be most accurate among persons with HIV in whom fungal burden is generally highest (*12*). Access to point-of-care, rapid antigen assays that do not require laboratory infrastructure would enable better characterization of histoplasmosis burden in limited resource settings, where the burden of advanced HIV disease is highest. Two companies, Optimum Imaging Diagnostics and MiraVista, have now produced lateral flow assays that have European Union CE marks, which indicates that a product has been assessed by the manufacturer and deemed to meet European Union safety, health, and environmental protection requirements; however, those products have not been approved by the US Food and Drug Administration (*7*,*12*,*13*). Another company, IMMY, is developing a lateral flow assay, but it has not yet been tested clinically. Further development and testing of these assays is necessary. Beyond the role of antigen testing to provide updated epidemiologic data, rapid antigen tests have the potential to improve clinical management among persons with advanced HIV disease and symptomatic histoplasmosis. If clinical trials demonstrate survival benefit from histoplasmosis screening and treatment among asymptomatic persons with advanced HIV disease, a rapid, point-of-care assay would be essential for diagnosis of cryptococcal diseases. 

Appendix Additional information about systematic review of prevalence of *Histoplasma* antigenuria in persons with HIV in Latin America and Africa.
