# Supplementary material for: Systematic Review of Prevalence of Histoplasma Antigenuria in Persons with HIV in Latin America and Africa
Source: Emerg Infect Dis. 2024 Aug;30(8):1523–30. doi: 10.3201/eid3008.231710 (PMC11286068; doi:10.3201/eid3008.231710)
Supplement: Appendix — Additional information about systematic review of prevalence of Histoplasma antigenuria in persons with HIV in Latin America and Africa. [file 23-1710-Techapp-s1.pdf]

Article DOI: https://doi.org/10.3201/eid3008.231710

EID cannot ensure accessibility for Appendix materials supplied by authors. Readers who have difficulty accessing Appendix content should contact the authors for assistance.

Systematic Review of Prevalence of *Histoplasma* Antigenuria in Persons with HIV in Latin America and Africa

Appendix

Appendix Table 1. Search criteria by search engines for systematic review of prevalence of *Histoplasma* antigenuria in persons with HIV in Latin America and Africa

Search	Description
Ovid, Medline, and epub ahead of print, in-process, in-data-review,and other nonindexed citations, daily and versions, 1946–July 20, 2023	
1	exp Histoplasmosis/ or exp Histoplasma/
2	(histoplasmosis or histoplasma or "h. capsulatum" or "h capsulatum").mp.
3	1 or 2
4	exp Antigens/ or exp Immunoenzyme Techniques/
5	(antigen* or enzyme immunoassay or enzyme immune assay or EIA).mp.
6	4 or 5
7	exp HIV Infections/ or exp HIV/
8	(AIDS or acquired immunodeficiency syndrome* or acquired immune deficiency syndrome* or acquired immuno-deficiency syndrome* or HIV or human immunodeficiency virus* or human immuno-deficiency virus).mp.
9	7 or 8
10	3 and 6 and 9
Embase Classic+Embase 1947–July 20, 2023	
1	exp Histoplasmosis/ or exp Histoplasma/
2	(histoplasmosis or histoplasma or "h. capsulatum" or "h capsulatum").mp.
3	1 or 2
4	exp antigen/ or exp enzyme immunoassay/
5	(antigen* or enzyme immunoassay or enzyme immune assay or EIA).mp.
6	4 or 5
7	exp Human immunodeficiency virus infection/ or exp Human immunodeficiency virus/
8	(AIDS or acquired immunodeficiency syndrome* or acquired immune deficiency syndrome* or acquired immuno-deficiency syndrome* or HIV or human immunodeficiency virus* or human immuno-deficiency virus).mp.
9	7 or 8
10	3 and 6 and 9
Scopus	
	((INDEXTERMS ("Histoplasmosis" OR "Histoplasma") OR TITLE-ABS-KEY (histoplasmosis OR histoplasma OR "h. capsulatum" OR "h capsulatum")) AND (INDEXTERMS ("Antigens" OR "Immunoenzyme Techniques" OR "antigen" OR "enzyme immunoassay") OR TITLE-ABS-KEY (antigen* OR enzyme AND immunoassay OR "enzyme immune assay" OR eia)) AND (INDEXTERMS ("HIV Infections" OR "HIV" OR "Human immunodeficiency virus infection" OR "Human immunodeficiency virus") AND TITLE-ABS-KEY (aids OR "acquired immunodeficiency syndrome*" OR "acquired immune deficiency syndrome*" OR "acquired immuno-deficiency syndrome*" OR “hiv*” OR "human immunodeficiency virus*" OR "human immuno-deficiency virus")))

Appendix Table 2. Agency for Healthcare Research and Quality (AHRQ) methodology checklist for cross-sectional/prevalence studies for systematic review of prevalence of *Histoplasma* antigenuria in persons with HIV in Latin America and Africa

Major components	Response options		
Define the source of information (survey, record review)	Y	N	U
List inclusion and exclusion criteria for exposed and unexposed subjects (cases and controls) or refer to previous publications	Y	N	U
Indicate time period used for identifying patients	Y	N	U
Indicate whether or not subjects were consecutive if not population-based	Y	N	U
Indicate if evaluators of subjective components of study were masked to other aspects of the status of the participants	Y	N	U
Describe any assessments undertaken for quality assurance purposes (e.g., test/retest of primary outcome measurements)	Y	N	U
Explain any patient exclusions from analysis	Y	N	U
Describe how confounding was assessed and/or controlled	Y	N	U
If applicable, explain how missing data were handled in the analysis	Y	N	U
Summarize patient response rates and completeness of data collection	Y	N	U
Clarify what follow-up, if any, was expected and the percentage of patients for which incomplete data or follow-up was obtained	Y	N	U

*U, unclear

Appendix Table 3. Risk of Bias Assessment Tool for Nonrandomized Studies (RoBANS)*

Domain	Description	Risk for bias
Selection of participants	Selection biases caused by the inadequate selection of participants	Low/High/Unclear
Confounding variables	Selection biases caused by the inadequate confirmation and consideration of confounding variables	Low/High/Unclear
Measurement of exposure	Performance biases caused by inadequate measurements of exposure	Low/High/Unclear
Blinding of outcome assessments	Detection biases caused by the inadequate blinding of outcome assessments	Low/High/Unclear
Incomplete outcome data	Attrition biases caused by the inadequate handling of incomplete outcome data	Low/High/Unclear
Selective outcome reporting	Reporting biases caused by the selective reporting of outcomes	Low/High/Unclear

Appendix Table 4: Risk of Bias Assessment Tool for Nonrandomized Studies (RoBANS) applied to cohort studies included in systematic review of prevalence of *Histoplasma* antigenuria in persons with HIV in Latin America and Africa*

Reference	Selection of participants	Confounding variables	Measurement of exposure	Blinding of outcome assessments	Incomplete outcome data	Selective outcome reporting
1	U	L	L	L	L	U
2	L	L	L	L	L	U
3	L	L	L	U	L	L
4	U	L	L	L	L	L

*U, unclear risk; L, low risk

Appendix Table 5. Agency for Healthcare Research and Quality (AHRQ) Methodology Checklist applied to cross-sectional studies included in systematic review of prevalence of *Histoplasma* antigenuria in persons with HIV in Latin America and Africa*

Ref	Info source defined	List inclusion/ exclusion	Time period indicated	Subjects consecutive	Blinded	QA	Patient exclusion from analysis explained	Confounding assessed	Missing data in analysis	Patient response rates and completeness of data	Followup for missing data
5	Y	Y	Y	Y	Y	U	Y	Y	Y	Y	U
6	Y	Y	Y	Y	U	U	Y	Y	NA	NA	NA
7	Y	Y	Y	Y	U	U	NA	Y	NA	NA	NA
8	Y	Y	Y	Y	U	Y	Y	U	NA	NA	NA
9	Y	Y	Y	Y	U	Y	NA	Y	NA	NA	NA
10	Y	Y	Y	Y	U	U	Y	U	NA	NA	NA
11	Y	Y	Y	Y	U	U	Y	U	NA	NA	NA
12	Y	Y	Y	Y	U	U	NA	Y	NA	NA	NA
13	Y	Y	Y	Y	U	U	Y	U	NA	NA	NA
14	Y	Y	Y	Y	Y	U	NA	Y	U	NA	NA
15	Y	Y	Y	Y	U	Y	Y	U	NA	Y	NA

*Ref, reference; Info, information; U, undetermined; NA, not applicable; QA, quality assurance

References

1. Vidal JE, Werlang PC, Muniz BM, Rego CM, Barbalho RE, Baptista AM, et al. Combining urine antigen and blood polymerase chain reaction for the diagnosis of disseminated histoplasmosis in hospitalized patients with advanced HIV disease. Med Mycol. 2021;59:916–22. PubMed https://doi.org/10.1093/mmy/myab022

2. Torres-González P, Niembro-Ortega MD, Martínez-Gamboa A, Ahumada-Topete VH, Andrade-Villanueva J, Araujo-Meléndez J, et al. Diagnostic accuracy cohort study and clinical value of the *Histoplasma* urine antigen (ALPHA Histoplasma EIA) for disseminated histoplasmosis among HIV infected patients: a multicenter study. PLoS Negl Trop Dis. 2018;12:e0006872. PubMed https://doi.org/10.1371/journal.pntd.0006872

3. Sekar P, Nalintya E, Kwizera R, Mukashyaka C, Niyonzima G, Namakula LO, et al. Prevalence of *Histoplasma antigenuria* among outpatient cohort with advanced HIV in Kampala, Uganda. J Fungi (Basel). 2023;9:757. PubMed https://doi.org/10.3390/jof9070757

4. Bahr NC, Sarosi GA, Meya DB, Bohjanen PR, Richer SM, Swartzentruber S, et al. Seroprevalence of histoplasmosis in Kampala, Uganda. Med Mycol. 2016;54:295–300. PubMed https://doi.org/10.1093/mmy/myv081

5. Schwartz IS, Kenyon C, Lehloenya R, Claasens S, Spengane Z, Prozesky H, et al. AIDS-related endemic mycoses in western Cape, South Africa, and clinical mimics: a cross-sectional study of adults with advanced HIV and recent-onset, widespread skin lesions. Open Forum Infect Dis. 2017;4:ofx186. PubMed https://doi.org/10.1093/ofid/ofx186

6. Ekeng BE, Oladele RO, Emanghe UE, Ochang EA, Mirabeau TY. Prevalence of histoplasmosis and molecular characterization of histoplasma species in patients with presumptive pulmonary tuberculosis in Calabar, Nigeria. Open Forum Infect Dis. 2022;9:ofac368.

7. Hoyos Pulgarin JA, Alzate Piedrahita JA, Moreno Gómez GA, Sierra Palacio JF, Ordoñez KM, Arias Ramos D. Closing gaps in histoplasmosis: clinical characteristics and factors associated with probable/histoplasmosis in HIV/AIDS hospitalized patients, a retrospective cross-sectional study in two tertiary centers in Pereira, Colombia. AIDS Res Ther. 2021;18:51. PubMed https://doi.org/10.1186/s12981-021-00377-5

8. Lofgren SM, Kirsch EJ, Maro VP, Morrissey AB, Msuya LJ, Kinabo GD, et al. Histoplasmosis among hospitalized febrile patients in northern Tanzania. Trans R Soc Trop Med Hyg. 2012;106:504–7. PubMed https://doi.org/10.1016/j.trstmh.2012.05.009

9. Ocansey BK, Otoo B, Asamoah I, Ganu V, Berko KP, Oladele O, et al. Cryptococcal and histoplasma antigen screening among people with human immunodeficiency virus in Ghana and comparative analysis of OIDx histoplasma lateral flow assay and IMMY histoplasma enzyme immunoassay. Open Forum Infect Dis. 2022;9:ofac277

10. Caceres DH, Zuluaga A, Arango-Bustamante K, de Bedout C, Tobón ÁM, Restrepo Á, et al. Implementation of a training course increased the diagnosis of histoplasmosis in Colombia. Am J Trop Med Hyg. 2015;93:662–7. PubMed https://doi.org/10.4269/ajtmh.15-0108

11. Caceres DH, Arauz AB, Flores C, Santiago E, Montoya S, Saenz C, et al. Implementation of rapid diagnostics assays for detection of histoplasmosis and cryptococcosis in Central American people living with HIV. Mycoses. 2021;64:1396–401. PubMed https://pubmed.ncbi.nlm.nih.gov/33966300

12. Oladele RO, Osaigbovo II, Akanmu AS, Adekanmbi OA, Ekeng BE, Mohammed Y, et al. Prevalence of histoplasmosis among persons with advanced HIV disease, Nigeria. Emerg Infect Dis. 2022;28:2261–9. PubMed https://doi.org/10.3201/eid2811.220542

13. Kuate MPN, Nyasa R, Mandengue C, Tendongfor N, Bongomin F, Denning DW. Screening for acute disseminated histoplasmosis in HIV disease using urinary antigen detection enzyme immunoassay: a pilot study in Cameroon. J Microbiol Methods. 2021;185:106226. PubMed https://doi.org/10.1016/j.mimet.2021.106226

14. van Schalkwyk E, Mhlanga M, Maphanga TG, Mpembe RS, Shillubane A, Iyaloo S, et al. Screening for invasive fungal disease using non-culture-based assays among inpatients with advanced HIV disease at a large academic hospital in South Africa. Mycoses. 2020;63:478–87. PubMed https://doi.org/10.1111/myc.13071

15. Edwards RJ, Todd S, Edwards J, Samaroo-Francis W, Lyons N, Boyce G, et al. The incidence of histoplasmosis and cryptococcal antigenemia among patients attending a large HIV clinic in Trinidad. Diagn Microbiol Infect Dis. 2023;106:115952. PubMed https://doi.org/10.1016/j.diagmicrobio.2023.115952
